# Understanding How Mental Health Influences IBD Outcomes: A Review of Potential Culprit Biological Mechanisms

**DOI:** 10.3390/biomedicines13122916

**Published:** 2025-11-28

**Authors:** Sherif Abdelbadiee, Giho Yoon, Kate Pearman, Aditi Kumar, Philip R. Harvey

**Affiliations:** Gastroenterology, The Royal Wolverhampton NHS Trust, Wolverhampton WV10 0QP, UKaditikumar@nhs.net (A.K.)

**Keywords:** inflammatory bowel diseases, Crohn’s disease, ulcerative colitis, anxiety, depression, Gut–Brain axis, hypothalamic–pituitary–adrenal axis, intestinal permeability, microbiota, dysbiosis, microbiome, serotonin, cortisol, stress, mast cells, cytokines, probiotics, antidepressive agents, psychotherapy, quality of life

## Abstract

Inflammatory bowel disease (IBD) includes Crohn’s disease (CD) and ulcerative colitis (UC). Similar to other chronic diseases, IBD is associated with negative mental health outcomes. The prevalence of anxiety and depression with IBD is increasing in western societies and there is a growing body of evidence suggesting a bidirectional relationship which remains poorly understood. This review seeks to distil current evidence on the epidemiology, biological mechanisms and microbial changes through which anxiety and depression may lead to worse IBD outcomes. The literature demonstrates that a prior diagnosis of depression is associated with an increased risk of developing IBD. Co-morbid anxiety or depression doubles the odds of adverse outcomes in IBD. Antidepressants appear to have class dependent effects on modulating disease activity in IBD with co-morbid depression. Chronic stress may drive IBD through a number of mechanisms, including inducing the hypothalamic pituitary axis, glucocorticoid resistance, increasing intestinal permeability, and releasing inflammatory cytokines. Alterations in the microbiome on either a genus or species’ level has been shown to be affected by and have an impact on both mental health illness and IBD activity. Further research with high quality longitudinal follow-up data is required to clarify causal associations of anxiety/depression and IBD onset as well as measure the impact of different antidepressant classes and microbiome targeted strategies on disease progression and outcomes.

## 1. Introduction

Inflammatory bowel disease (IBD) is a chronic, inflammatory condition of a relapsing–remitting nature affecting the gastrointestinal (GI) tract. It presents in two primary forms; Crohn’s disease (CD) and ulcerative colitis (UC), which, although closely related, have key phenotypic and aetiological differences. Despite significant advances in basic science [1], their exact aetiology and factors influencing severity and prognosis remain unclear. It is widely thought to be an inflammatory, immune mediated disorder, occurring in those with a genetic predisposition, further associated with exposure to various environmental and lifestyle factors such as Western diets, smoking, urban living, early life antibiotic exposure, gastrointestinal (GI) microbiome alterations and surgeries such as appendectomy and tonsillectomy [2,3,4].

Mental health disorders, particularly anxiety and depression, are increasingly associated with chronic physical illnesses [5,6]. Anxiety is characterised by an inordinate, persistent fear and apprehension towards a perceived future threat whereas depressed mood is defined by feelings of hopelessness, sorrow and feeling void [7]. The latter is one of two essential diagnostic criteria for the diagnosis of Major Depressive Disorder (MDD) that must be present for at least two weeks [7,8].

Given the chronic, relapsing nature of IBD and its substantial physical and psychosocial burden; individuals with IBD are susceptible to developing anxiety and depression [9]. There is an accumulating body of evidence towards a more bidirectional relationship between these disorders. Two recent systematic reviews and meta-analyses demonstrated that there is an increased propensity in individuals diagnosed with depression to later develop IBD, with diagnoses of depressive mood disorders predating IBD diagnosis by more than 5 years [9,10]. These findings indicate potential causality associations between depression and the future onset of IBD. Given the prevalence of anxiety and depression and their bidirectional relationship with IBD, it is important to consider these disorders in the management, progression and possibly prevention of IBD.

This review article aims to describe the biologically plausible mechanisms by which anxiety and depression might influence IBD outcomes. We also present epidemiological data of anxiety and depression in IBD patients and associated variations in outcomes.

## 2. Epidemiology of Anxiety and Depression in IBD and Associated Influence on Outcomes

In western populations approximately 20% of men and 30% of women experience depression in their lifetime. A 2016 systematic review reported a 25% prevalence of anxiety disorders in the general population [11,12]. A longitudinal cohort study including 388 patients with IBD observed an anxiety or depression diagnosis predated a diagnosis of IBD, in more than half the patients, by more than 2 years [13]; suggesting potential causality.

Both co-morbid anxiety and depression are associated with worse outcomes, even in IBD patients who are in clinical remission. Studies have shown their presence increased the relative risk (RR) of poor IBD outcomes including rates of disease flare (RR 1.6), steroid resistance (45.5% vs. 23.8%), need for therapy escalation (RR 1.41), emergency department visits (RR 1.3), hospital admissions (RR 1.35), and surgery (RR 1.63); posing a further challenge in the management of disease flare ups [14,15,16].

A cross-sectional study of over 340 patients with IBD showed co-morbid anxiety/depression was associated with higher endoscopic disease severity scores. In UC, the median Mayo scores were higher in patients with concurrent anxiety/depression symptoms than those without (Mayo score 10 vs. 8, respectively), median ulcerative colitis endoscopic index of severity (UCEIS) scores (UCEIS 6 vs. 4, respectively) and surgery rates were also higher (22.7% vs. 5.3%, respectively). In CD median Crohn’s disease activity index (CDAI) was significantly higher in patients with symptoms of anxiety/depression than those without (CDAI 356.0 vs. 189.5, respectively), higher median simple endoscopic score for Crohn’s disease (SES-CD) (13 vs. 12, respectively) and similarly, surgery rates were higher (56.5% vs. 41.5%, respectively). CRP and ESR Laboratory markers were also higher in CD patients with these comorbidities, though findings were inconsistent in UC [17].

The recent British Society of Gastroenterology (BSG) IBD guidelines published in 2025 highlight that pain is a more frequently reported symptom in those suffering with anxiety or depression [18]. They recommend screening for psychological causes in patients reporting pain and suggest that mental health therapies may be offered as adjuncts in disease management. A 2021 study which screened IBD patients for mental health concerns of significance using the Hospital Anxiety and Depression Scale [HADS] and the Kessler 6 Scale [K6]; showed patients scoring above clinical cut off values, who accepted psychological intervention, reported better mental and total quality of life outcomes at 12 months compared to those who declined [19]. Frolkis et al.’s 2019 retrospective cohort study showed reduced hazard ratios (HR) of new onset IBD were associated with anti-depressant use [20]. The data also demonstrated that selective serotonin reuptake inhibitors (SSRI) and tricyclic antidepressants (TCA) were more protective against new onset CD (HR 0.63 vs. 0.77, respectively) than UC (0.48 vs. 0.59, respectively). Serotonin-norepinephrine reuptake inhibitors (SNRIs) and Mirtazapine were selectively protective against UC (HR 0.46 vs. 0.34, respectively). This implies distinct mechanisms linking mental health disorders and the IBD subtypes [20].

In the last quarter century, the incidence of IBD has doubled in patients younger than 18 years old [21]. Given the prevalence of anxiety and depression in society, improving our understanding of the pathogenesis of IBD as well as its contributing factors (including these mental health disorders) is crucial. This knowledge may help us identify new therapeutic targets for the management and potentially even prevention of IBD.

## 3. Biological Mechanisms Linking Anxiety, Depression and IBD

**Figure 1:**
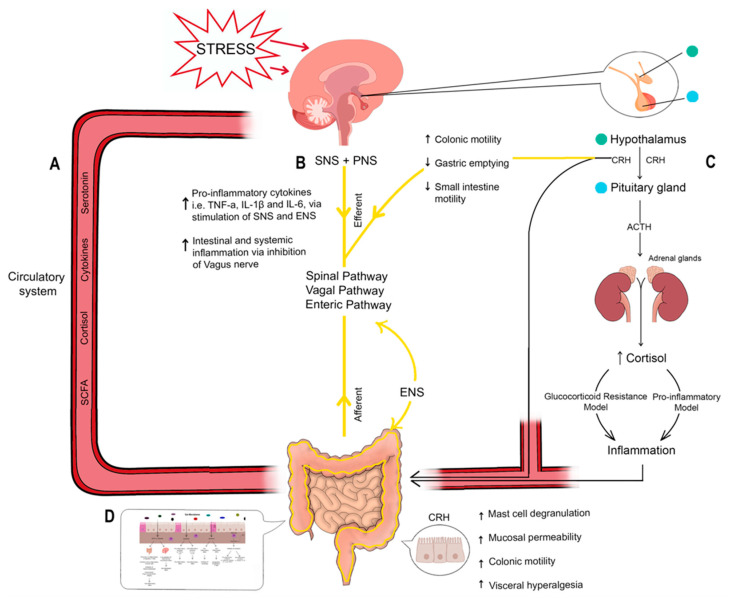
Illustration of the different systems linking the brain and gut when stress is encountered. (**A**) Outlines the circulatory system transporting components between the gut, central nervous system (CNS) and neuroendocrine system. Components such as cortisol, cytokines, serotonin and short-chain fatty acids (SCFAs) play a role in IBD as well as anxiety/depression. (**B**) Outlines the Gut–Brain Axis and Autonomic nervous system (SNS + PNS + ENS). It illustrates the efferent and afferent signals linking the CNS and gut via spinal, vagal and enteric pathways. The ENS within the gut is labelled separately but demonstrates links to the SNS and PNS, highlighting its ability to function independently as well as alongside the other components of the ANS. When the body encounters stress, there is an increase in pro-inflammatory cytokines TNF-α, IL-1β and IL-6 facilitated by the SNS and ENS. In addition, the inhibition of the vagus nerve further contributes to the increased inflammation seen. (**C**) Outlines the effect of stress on the neuroendocrine system and the subsequent effect on the gut. There is a focus on the HPA axis whereby stress triggers the release of CRH from the hypothalamus. Acting via the ANS, CRH increases colonic motility whilst decreasing gastric emptying and small intestine motility in the GI system. Peripheral CRH causes increased mast cell degranulation, mucosal permeability, colonic motility, and visceral hyperalgesia. CRH release in the hypothalamus in turn stimulates ACTH release from the pituitary gland. ACTH stimulates cortisol release from the adrenal glands, which exhibits an inflammatory effect on the gut. The method by which cortisol causes this inflammatory effect is debated and is described by the ‘glucocorticoid resistance’ model and the ‘pro-inflammatory’ model, respectively. The sum of these effects contributes to the GI symptoms seen following stress. (**D**) Miniaturisation of Figure 2. Provides an overview of the effects of microbial metabolites, serotonin, mast cells, and cytokines on inflammation. See Table 1 and Figure 2 for more details.

The Gut–Brain Axis (GBA) is a bi-directional system between the autonomic nervous system (ANS), the gut and our neuro-endocrine system with numerous inter-links at work (Figure 1) This section will explore how the GBA and the microbiome link these conditions [22,23,24].

### 3.1. Hypothalamic–Pituitary–Adrenal Axis and Cortisol

Psychological stress activates the hypothalamic pituitary (HPA) axis stimulating the release of corticotrophin releasing hormone (CRH) from the hypothalamus. This stimulates adrenocorticotrophic hormone (ACTH) release from the pituitary gland and subsequent cortisol secretion from the adrenal glands [25]. Depression and anxiety are associated with HPA axis hyperactivity and CRH release [26,27,28].

A trademark characteristic of depression is thought to be a pro-inflammatory state, alongside hypercortisolism [29,30]. Although cortisol mainly has immunosuppressive properties [31], its chronic elevation may lead to ‘glucocorticoid resistance’; whereby immune cells’ glucocorticoid receptors demonstrate a reduced sensitivity to cortisol, leading to compromised inhibition of inflammation. This model explains the paradoxical inflammation despite elevated cortisol levels [32,33]. Another ‘pro-inflammatory cortisol’ model exists, suggesting that the increased inflammation can be attributed to cortisol itself having a pro-inflammatory effect [30].

The association of cortisol with GI inflammation is supported in part by an animal study in which mice were treated with a glucocorticoid receptor (GR) agonist—dexamethasone—at a concentration mimicking corticosterone levels seen in the serum during stress. They were then exposed to dextran sodium sulphate (DSS) to induce colitis. This dual exposure resulted in an early mortality, reduction in colonic length, colonoscopic and histological evidence of mucosal damage, and overall, a more severe disease presentation. In addition, effects of stress on the gut were shown to be mitigated through a reduction in corticosterone via various means (CRH Receptor 1 antagonist, adrenalectomy or GR inhibition), preventing aggravation of colitis [34].

A similar argument to the ‘glucocorticoid resistance’ model is made for the relationship of cortisol with anxiety [27]. Ultimately, it is conceded that these mechanisms require further research to establish clarity.

### 3.2. Corticotrophin Releasing Hormone and Mast-Cell Activation

In addition to cortisol, when considering the effects of the HPA axis on inflammation, CRH must not be overlooked. CRH is a key component of the hypothalamic–pituitary–adrenal (HPA) axis [35]. It is expressed centrally through the HPA axis and peripherally in the enteric system, with the action of CRH and CRH receptors varying between these sites [36,37]. The ANS facilitates the action of brain CRH which has contradicting effects on the upper and lower digestive tract. It reduces gastric emptying and inhibits small intestine motility, whilst stimulating colonic motility and bowel movement [38]. CRH acting peripherally increases mucosal paracellular permeability, mast cell degranulation, colonic motility, and visceral hyperalgesia [38,39,40]. See Figure 1C.

The effect of stress on intestinal permeability has been evidenced in an animal study which showed that depressed rodents (provoked via maternal separation) displayed increased intestinal permeability and were more susceptible to induced colitis, as well as having a more severe presentation [41]. A 2013 human study provided evidence that psychological stress—conveyed in the form of public speaking—led to increased small intestinal permeability in participants whose salivary cortisol levels had risen above the 90th percentile as a result of this acute stressor [39]. This effect was replicated through the peripheral administration of CRH, suggesting the underlying mechanism through which stress induced activation of the Brain–gut axis increases intestinal permeability. It is important to note that the type of stress induced in this study is of an acute nature, with short lasting effects. It would not be ethically feasible to replicate chronic stress when designing human studies and therefore this poses a challenge for studying the long term impact of chronic stress on intestinal permeability [42].

CRH stimulates immune cells such as mast cells in the gut, triggering the release of pro-inflammatory cytokines including TNF-α (a Target of anti-TNF therapies) and IL-6 (akin to the effect of nicotine increasing IL-6 in the small bowel, contributing to mucosal damage) [43]. These processes disrupt mucosal barrier integrity and alter tight junction proteins such as occludin, leading to increased intestinal permeability, commonly referred to as leaky gut [39,44]. CRH receptor antagonists have demonstrated efficacy in blocking this effect in ex vivo animal studies [45], though human in vivo studies assessing their effect are still lacking.

Mast cells and their mediators play a pivotal role in IBD. Their accumulation and degranulation is commonly seen in both UC [46,47] and CD [46,47,48]. Certain mast cell mediators (histamine, tryptase, IL-1, IL-6, TNF-α) contribute to inflammation seen in IBD [46,49]. Dysregulation of various chloride channels expressed in mast cells also plays a role in the inflammation and epithelial barrier dysfunction seen in IBD. These channels include Chloride channel-2 (ClC-2) and Cystic Fibrosis Transmembrane Conductance Regulator (CFTR). ClC-2 channels help regulate mast cell volume and mediator release. CFTR channels, on the other hand, may aid the process of degranulation and mediator release from mast cells. Chloride channel dysregulation has been associated with IBD, leading to a negative impact on intestinal barrier function and permeability, immune modulation and an increased release of pro-inflammatory mediators [49].

**Figure 2:**
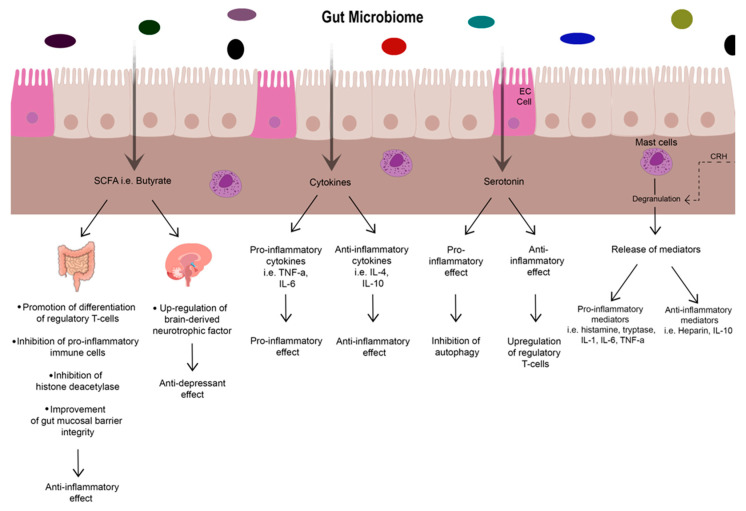
Illustration of gut microbiome and description of the effects of microbial metabolites, serotonin, cytokines, and mast cells on inflammation. The prevalence of bacteria in the **gut microbiome** varies in different conditions and this in turn can impact the levels of microbial metabolites, serotonin, and cytokines. Table 1 explores certain bacteria and their association with anxiety/depression, as well as IBD. Certain bacteria such as *F. prausnitzii* produce the **short chain fatty acid, Butyrate.** Butyrate has anti-inflammatory effects on the gut via inhibition of pro-inflammatory immune cells and histone deacetylase, whilst promoting regulatory T-cell differentiation and gut mucosal barrier integrity. It also exerts an anti-depressant effect centrally by stimulating formation of brain-derived neurotrophic factor (BDNF). **Cytokine** production stimulated by different bacteria play either pro or anti–inflammatory roles. Examples in this figure include TNF-α and IL-6 as pro-inflammatory in nature, whilst IL-4 and IL-10 are anti-inflammatory. **Serotonin** is predominantly produced by enterochromaffin cells. Serotonin is also produced by certain species of bacteria in the microbiome. It is seen to display a dual role in the gut. It can display pro-inflammatory effects as higher concentrations of serotonin increase susceptibility to colitis via inhibition of autophagy. Paradoxically, exposure to serotonin producing gut microbiota earlier in life can improve tolerance to dietary antigens and gut commensals by upregulating regulatory T-cells, which has an anti-inflammatory effect. **Mast cells** stimulated by CRH peripherally degranulate to release mediators including cytokines, which are similarly varied in pro and anti-inflammatory effects.

Other mast cell mediators (heparin and IL-10), however, are anti-inflammatory and may represent potential therapeutic targets [46]; see Figure 2. As such, it is important to also recognise that despite the pro-inflammatory characteristics mast cells have, they do also seem to play a protective role in the context of IBD and gut health. In a study investigating their role in IL-10 deficient mice, they were shown to display a protective effect through optimisation of mucosal health, as their absence in IL-10 deficient mice predisposed to spontaneous IBD [50]. The pathophysiology and mechanisms behind each of these mediators in the context of IBD is complex and beyond the scope of this review but can be found well summarised by He (2004) [46].

### 3.3. Autonomic Nervous System

The autonomic nervous system (ANS) consists of the sympathetic nervous system (SNS) and parasympathetic nervous system (PNS), as well as the enteric nervous system (ENS), and holds a vital role in body homeostasis and controlling systemic responses [51]. Utilising enteric, vagal and spinal pathways, the SNS and PNS both communicate afferent signals from the lumen to the CNS, as well as transmitting efferent signals from the CNS to the GI system. The SNS controls circulation and inhibits gastric musculature and mucosal secretion. The PNS provides inhibitory as well as excitatory control over gastrointestinal function [51,52].

The ENS is often thought of as the ‘brain in the gut’ and has a major role in GI circulation, immune function, motility, and secretion. Whilst the ENS communicates with the central nervous system, it is able to function independently [53,54]. It comprises numerous neurons and glial cells which are grouped into two major ganglionated plexuses—the submucosal and myenteric plexus [55,56]. The submucosal plexus is located in the connective tissues of the submucosa and is prominent primarily in the small intestine, whereas the myenteric plexus is located between the longitudinal and circular layers of the whole GI tract [57,58].

The ANS, as a whole, responds to psychological stress caused by anxiety and depression leading to notable GI consequences. The SNS, when triggered by stress, causes the release of adrenaline and noradrenaline which facilitates an increase in pro-inflammatory cytokines such as TNF-α, IL-1β and IL-6 [54,59]. See Figure 1B. This is compounded by increased intestinal and systemic inflammation caused by the inhibition of the vagus nerve [25]. In the context of extended stress-as is in the case of anxiety and depression-there is persistent activation of the SNS and inhibition of PNS increasing circulating pro-inflammatory cytokines [59]. The ENS is also affected by such changes in the GBA. As explored previously, prolonged stress can elevate glucocorticoid levels. One study suggests that this stimulates production of an inflammatory phenotype of enteric glia called **e**nteric **g**lia **a**ssociated with **p**sychological **s**tress (eGAPS) which promotes inflammation through Colony Stimulating Factor-1 (CSF-1) [34]. In this process, there is a build-up of monocytes which produce TNF, subsequently promoting a pro-inflammatory picture. This is the target of anti-TNF drugs such as infliximab for IBD. These were some of the first biologic therapeutic agents for the treatment of IBD and remain among the most effective [60,61]. In addition, elevated glucocorticoids also reduce colonic transit time by affecting the maturity of enteric neurons. The cumulation of these factors highlights the compounded effect of ANS on worsening IBD and exacerbating GI symptoms [34].

### 3.4. Immune System and Cytokines

Cytokines are instrumental in regulating intestinal inflammation and complications in IBD patients. Anxiety and depression are linked to increased levels of pro-inflammatory cytokines such as TNF-α and IL-6 as mentioned earlier [62,63,64,65]. Anxiety has also been associated with lower levels of anti-inflammatory cytokines such as IL-4 and IL-10 [65,66]. By comparison, a meta-analysis investigating cytokines in major depression has found no significant difference in levels of IL-4 and IL-10 in those depressed compared to those who were not, though the potential limitation of a small sample size should be noted [64]. This highlights the variation in possible mechanisms by which anxiety and depression may affect the inflammatory process. The relationship between the immune system, anxiety/depression, and IBD is important to acknowledge, especially given the immune systems role as a key therapeutic target [62].

## 4. The Gut Microbiome

Recent research into the pathophysiology of IBD has described alterations of the gut microbiome as a potential precursor to the underlying inflammatory response [67,68,69,70]. This alteration of the gut microbiome has also been implicated in mental health disorders such as anxiety and depression [71,72,73,74,75,76]. Evolving research implicated the microbiome as a key component in various inflammatory diseases, such as IBD and Systemic Lupus Erythematosus [77,78,79]. This section discusses how the gut microbiome composition, microbiome metabolites making up the gut metabolome (e.g., short chain fatty acids) and serotonin mediated neuromodulation may play a role in IBD, anxiety and depression. A visual summary of some of these effects is shown in Figure 2.

### 4.1. Neuromodulation, Serotonin and Vascular Barriers

95% of serotonin is primarily synthesised in the GI tract by enterochromaffin (EC) cells. Its pathway utilises an enzyme called TPH1 which converts dietary tryptophan to 5-hydroxy-L-tryptophan (5-HTP), which is later converted to serotonin [80]. TPH1 deficiency thus leads to low serotonin levels. Low serotonin has been implicated in anxiety and depression and is a target for medication such as SSRIs that increase the level of circulating serotonin [81].

Serotonin in the gut regulates intestinal motility and secretions. Alterations in serotonin regulation have been associated with functional bowel disorders and IBD, as well as having an effect on almost all human behaviours including mood and aggression amongst others [80,82]. It appears to have a dual role in inflammation too. Firstly, it can exhibit pro-inflammatory effects on the gut and is associated with worse colitis severity [80,83,84,85]. It has an inhibitory effect on the cellular recycling mechanism of autophagy in the gut, inhibiting autophagy’s seemingly protective role against colitis [80,86]. See Figure 2. TPH1-deficient mice who had their serotonin levels replaced through subcutaneous administration of 5-HTP, had an increased susceptibility to dextran sulphate sodium induced colitis [80]. Disturbances with autophagy at the level of the genome (ATG16L1/IRGM) are thought to have a role in the pathogenesis of IBD [86,87,88,89]. Therefore, a similar link may be through serotonin’s inhibition of autophagy in the gut.

Conversely, a 2024 study by Sanidad et al. [90] showed that early life exposure to serotonin-producing gut microbiota could have anti-inflammatory effects. This is thought to be mediated through improved tolerance to dietary antigens and gut commensals by serotonin stimulating formation of regulatory T-cells [90]. This protective effect appeared to be age-dependent with a lack of replicated effect in adult mice exposed to orally administered serotonin (although only administered for two days). Monoamine oxidase A (MAO-A) enzyme breaks down serotonin into 5-hydroxyindoleacetic acid. It was found to be 15 times lower in the small intestine of neonatal mice raised in specific pathogen-free (SPF) conditions. In addition, the majority of serotonin production in the neonatal intestine was found to be directly driven by gut bacteria through direct production, the upregulation of TPH1 as well as suppression of MAO-A. This was reflected by much higher serotonin levels in the small intestine and colonic luminal contents of SPF mice compared to germ-free (GF) mice. Thus, it could be theorised that prolonged SSRI exposure (as in Frolkis et al.’s 2019 paper [20]) is protective against IBD via similar mechanisms to the early life exposure to serotonin producing microbiota. This dual role of serotonin in the gut is an area requiring further research. This anti-inflammatory effect is shown in Figure 2.

GF mice were also noted to have significantly lower levels of the tight junction proteins (occludin and claudin-5) in their blood-brain barriers (BBB). These proteins are essential to BBB integrity and regulation of its permeability. Colonisation of GF mice with gut microbiota from pathogen-free mice resulted in the up-regulation of both these proteins and improved BBB integrity [91]. The recent literature has described that the gut-vascular barrier (GVB) and the BBB share a similar architecture and that alternation in claudins could lead to a loss of barrier integrity across both the GVB and BBB [92]. Gut microbiome alterations have also been found to lead to the disruption of both the GVB and the BBB [91,93]. These disruptions could increase susceptibility to neuroinflammation through the translocation of pro-inflammatory cytokines IL-6, IL-1β and pro-inflammatory High-mobility group box 1 (HMGB1) proteins elevated in IBD. Elevated faecal levels of HMGB1 proteins are found in IBD as a result of cell injury. IL-6, IL-1β and HMGB1 were shown to be elevated in the hippocampus of mice with DSS-induced chronic colitis [94,95]. Hippocampal injury and resultant volume loss is known to be associated with a higher number of depressive episodes in individuals [96,97]. These mechanisms may provide a further explanation for depression seen with inflammatory bowel disease.

Various species of gut bacteria, such as *Lactobacillus plantarum*, produce and interact with neurotransmitters such as serotonin, noradrenaline and dopamine [98,99]. They also possess receptors for varying neurotransmitters; however, this relationship is not well understood. Certain strains cause upregulation of TPH1 [90] while others such as *Lactobacillus acidophilus* have been shown to up-regulate serotonin transporter (SERT) expression-involved in serotonin clearance, thus leading to reduced serotonin bioavailability [100].

### 4.2. Microbial Metabolites: Short Chain Fatty Acid (SCFAs)

Certain gut microbiota produce metabolites such as butyrate, acetate and propionate which are short chain fatty acids (SCFAs), some of which have anti-depressant effects. Reigstad et al. found gut microbiota and SCFAs metabolites influenced enteric serotonin production and homeostasis [101]. In mouse models, the SCFA butyrate, up-regulated brain-derived neurotrophic factor (BDNF) production which possesses antidepressant effects. It is also associated with increased expression of occludin in the BBB, improving its integrity and reducing permeability [91]. Butyrate also exerted anti-inflammatory effects on the bowel through various mechanisms including promoting differentiation of regulatory T cells, inhibiting pro-inflammatory immune cells, inhibition of histone deacetylase and improving the integrity of the gut mucosal barrier [102]. See Figure 2.

**Table 1:** This table summarises gut microbiota (genus/species) and the discussed association with anxiety/depression and IBD. *Variable* is used when research is inconsistent, either between studies or species.

Bacteria (Genus/Species)	Prevalence in Anxiety/Depression	Prevalence in IBD	Association with IBD Activity and Anxiety/Depression	Notes
*Escherichia coli*	Increased	Increased	Pathogenic strains are associated with increased activity of CD/UC Colibactin-producing *E. coli* associated with anxiety and depression-like behaviours	Pathogenic Strains, e.g., AIEC associated with worse IBD.
*Faecalibacterium*	Decreased	Decreased	*F. prausnitzii*—associated with reduced activity of CD/UC and reduction in anxiety-like and depression-like behaviour	Role in SCFA Production. Reduces serotonin degradation leading to increased levels of serotonin in the gut
*Lactobacillus*	Decreased	Variable ^1^	Associated with reduced activity of UC-Pre and probiotic administration improved symptoms in UC Certain strains associated with anti-depressant and anxiolytic effects, e.g., L. *plantarum 286*	Reduced pro-inflammatory mediators and cortisol. Role in serotonin Synthesis (1) Increased in active CD, but appears protective in UC
*Clostridioides*	Variable	Variable	*C. butyricum*—associated with reduced activity of UC *C. leptum* reduction is associated with increased depression severity *C. difficile* is associated with depression/anxiety-like behaviours.	*C. butyricum*—associated with SCFA production and increased serotonin
*Bacteroidetes*	Variable ^2^	Decreased	*B. vulgatus*—Associated with reduced activity of both CD/UC + anxiety/depression	(2) Conflicting effects between disease groups.
*Bifidobacterium*	Decreased	Decreased	*B. longum*—associated with reduced activity of both CD/UC and lower depression scores.	Role in SCFA Production, Tryptophan metabolism
*Roseburia*	Variable	Decreased	Associated with reduced activity of both CD/UC Depletion of *Roseburia* is associated with MDD	SCFA Production Conflicting research in MDD. May be involved in serotonin expression.

Abbreviations: SCFA = Short-chain fatty acids, IBD = Inflammatory Bowel Disease, AIEC = adherent-invasive *E. coli*.

### 4.3. Escherichia coli (E. coli)

IBD variations in gut microbiota show a high Abundance of *E. coli* in mucosal biopsies and stool specimens. Many strains isolated in active IBD are pathogenic such as adherent-invasive *E. coli* (AIEC) [79]. These strains are thought to display virulence through attachment, invasion and toxin production, all of which have been implicated in the pathogenesis of IBD. A gut where inflammation and mucosal disruption is present creates favourable conditions for these pathogenic properties. Studies have also highlighted that preceding gut microbiota changes such as changes in overall diversity and gut permeability, as well as changes seen in IBD, makes infection with AIEC more likely [103,104,105]. Gut microbiota alterations and inflammation have also been shown in patients with a diagnosis of anxiety/depression. Pathogenic *E. coli* strains have been found to be influenced by stress-related effects seen in anxiety as well as a higher prevalence in those with diagnosed generalised anxiety disorder (GAD) [106,107]. Colibactin producing strains were also associated with increased anxiety and depression-like behaviour in mice [108].

Jang et al. used immobilisation stress (IS) in mice to promote anxiety and examined for alterations in the microbiome [104]. This resulted in a significant increase in *Enterobacteriaceae* populations with a higher abundance of pathological *E. coli* and a reduction in *Lactobacillus* at the family level; further supporting the role of stress in altering the gut microbiome. The faecal microbiota of these IS-exposed mice was then fed to germ-free mice. They observed an increase in anxiety-like behaviours and features of colitis including colon shortening, increased Myeloperoxidase (MPO) activity and Tumour Necrosis Factor (TNF) Alpha expression. They then purified lipopolysaccharide (LPS) from *E. coli* and injected this intraperitoneally into the germ naive mice. This again caused anxiety behaviour with induced activation of IL-6, TNF-α and IL-1β expression, indicating colitis.

This supports the hypothesis that stress and anxiety states, enriched with an abundance of pathogenic AIEC and colibactin-producing strains, could in turn promote gut inflammation and potentially be implicated in IBD, given the cellular effects described. However, it is unclear whether pathogenic bacteria like AIEC are causing intestinal inflammation that leads to the incidence of disease or whether this increased prevalence in inflamed gut microbiomes leads to worsening of disease [79].

### 4.4. Faecalibacterium prausnitzii (F. prausnitzii)

*F. prausnitzii* is one of the most abundant bacterial species in a healthy human gut [109,110]. It is a short-chain fatty acid (SCFA) producing bacterium which has anti-inflammatory properties as a main butyrate producer within the GI system. Butyrate plays a role in gut homeostasis by decreasing intestinal inflammation at the level of the mucosa through inhibition of Nuclear Factor kappa-light-chain-enhancer of activated B cells (NF-κB) and interferon-gamma (IFN-γ) [109,111,112,113,114]. It also stimulates the secretion of protective cytokines such as IL-10 [115]. Studies have shown that reduced levels of *F. prausnitzii* in patients with CD and UC has been associated with increased levels of inflammation and higher disease severity [110].

Samuthpongtorn et al. found patients with depression had reduced levels of the S-adenosyl-L-methionine (SAM) cycle I pathway, a pathway that is performed by *F. prausnitzii* leading to reduced expression of (MAO-A), which is instrumental in the breakdown of neurotransmitters such as serotonin and dopamine [116]. Abundance of *F. prausnitzii* could therefore lead to reduced degradation of serotonin and this was further supported by Martin et al., where treatment with *F. prausnitzii* led to increased intestinal serotonin levels in murine models [117]. Hao et al. found consumption of *F. prausnitzii* reduced anxiety and depression-like behaviours in rats [118]. Lower levels of *F. prausnitzii* have also been seen in psychiatric disorders such as anxiety and depression [76]. Another study showed social exclusion, a cause of anxiety, was associated with reduced abundance of *Faecalibacterium* [119].

Thus, the lower levels of *F. prausnitzii* seen in anxiety and depression could decrease their anti-inflammatory mediators in the gut. This may then contribute to heightened disease severity and inflammation seen in IBD. These effects have only been demonstrated in animal models; therefore, further human studies are needed to evaluate this further.

### 4.5. Lactobacillus

*Lactobacilli* are Gram-positive facultative anaerobic bacterium. A meta-analysis and systematic review found treatment with *Lactobacillus*-containing probiotics and pre-biotics had a significant positive effect on patients with UC [120].

Conversely, other studies have found abundance was increased in CD, and increased levels of *Lactobacillus* correlated with a depletion of the protective bacterium *F. prausnitzii* [121,122,123]. Specific probiotic strains like *Lactobacillus plantarum* (*L. plantarum*) have shown to increase serotonin pathway gene expression, with ingestion in mice models significantly increasing Tryptophan Hydroxylase (TPH1) [124]. Although peripheral serotonin cannot cross the BBB, it can interact with the ENS which is a mechanism for further exploration.

A study looking at the microbiome in patients with UC and a diagnosis of anxiety/depression found a significant decrease in *Lactobacillaceae* when compared to patients with UC without anxiety/depression [125]. An animal study using *L. plantarum 286* showed it was associated with anti-depressant and anxio-lytic effects in the mice in which it was administered [126]. *Lactobacillus* has been shown to decrease pro-inflammatory mediators implicated in IBD such as IL-6, TNF-α and IL-1β [127].

It is still unclear the exact role of this species and its relationship with IBD and mental health disorders. This lack of clarity is in part, due to differing effects at strain level. Further research is also needed into the role of these disease groups on *Lactobacillus*.

### 4.6. Clostridioides

*Clostridioides* is an anaerobic Gram-positive genus. *Clostridioides difficile* (*C. difficile)* is the most prevalent cause of antibiotic-associated colitis [128,129]. Patients with IBD are not only at higher risk of this infection but have been shown to have poorer outcomes with higher instances of colectomy and mortality [129]. *C. difficile* has also been shown to increase depression and anxiety-like behaviours in mice with increased levels of IL-6 both in the colon and hippocampus [130]. Studies found an increased prevalence of *Clostridioides* in mice exposed to social stressors, as well as a positive correlation of *Clostridioides* with negative social behaviours [131,132].

*Clostridioides butyricum* (*C. butyricum*) is one strain that is commonly used in probiotic formulations, likely due to its anti-inflammatory effects associated with SCFA production. One study looked at how it affects depression-like behaviour in stress-induced mice models [133]. Mice treated with *C*. *butyricum* showed less depression-like behaviour as well as an increased abundance of serotonin. This species has also been associated with lower disease activity in patients with UC [134]. Low levels of another species—*C. leptum*—have been shown to be associated with higher depression scores in a 2019 cross-sectional, observational study of 75 participants [135].

Therefore, this genus exerts varying effects dependent on species, with species like *C. butyricum* having protective anti-depressant and anti-inflammatory effects, whilst *C. difficile* is associated with depression and anxiety-like behaviours.

### 4.7. Bacteroidetes

*Bacteroidetes* are Gram-negative anaerobic bacteria prevalent in the human gut. A 2016 meta-analysis found lower levels of *Bacteroidetes* in the gut microbiome of patients with IBD, especially those with active disease compared to healthy individuals [136]. Zhang et al. found transplanting faecal microbiome from humans with major depressive disorder (MDD) into mice induced colonisation with *Bacteroidetes* and produced anxiety and depression-like behaviours [137]. The behavioural assessment was carried out using the open-field test, social interaction test, tail suspension test and elevated plus maze test. These tests examined movements and sociability to deduce anxiety and despair-like behaviours. The main species implicated with these behaviours were *B. fragilis*, *B. uniformis* and *B. caccae* to a smaller degree. Hu et al. also found increased levels of *Bacteroidetes* in both moderate and severe MDD [138].

Conversely, specific strains such as *Bacteroidetes vulagtus* (*B. vulgatus*) have demonstrated a protective effect both in IBD and IBD with comorbid depression. Wu et al. found lower levels of *B. vulgatus* in the patients with CD and UC who also had depression. In murine models, mice pretreated with *B. vulgatus* showed lower disease activity as well as an alleviation of depression-like behaviour, thus suggesting an association between reduced abundance of this strain, depression and worse colitis. This combined effect was thought to be due to the predominant metabolite of *B. vulgatus*; p-hydroxyphenylacetic acid (4-HPAA) [139]. 4-HPAA is a product of gut microbiota breakdown of dietary phenols and is thought to be involved in gut homeostasis [140]. Shao et al. demonstrated that in vitro that administration of 4-HPAA increased intestinal barrier integrity and had antioxidant effects [141].

Overall, the evidence for the *Bacteroidetes* phylum is conflicting and may relate to specific species and their metabolites. Certain species may demonstrate a shared mechanism between disease activity in IBD and mental health as with the protective effects of *B. vulgatus*. Therefore, further research at species level is warranted.

### 4.8. Bifidobacterium

*Bifidobacterium* is a Gram-positive bacterium with over 50 different species [142]; many produce SCFAs and reduce inflammatory cytokines implicated in IBD [143]. One of the most abundant species is *Bifidobacterium longum* (*B. longum*) [142]. *B. longum* can improve inflammation and symptoms associated with colitis [144,145,146]. A human trial also demonstrated reduced depression scores in patients who received probiotic *B. Longum,* although this effect was not seen on anxiety scores [147]. Furthermore, a recent systematic review looking at gut microbiota found lower levels of this genus in patients with anxiety/depression [148].

*B. longum* is also involved in tryptophan metabolism [149]. Tryptophan, as previously mentioned, is a precursor to serotonin as part of the serotonin pathway and also kynurenine (KYN) as part of the KYN pathway. Levels of metabolites of these pathways have been shown to be different in patients with CD and co-morbid depression compared to those with only CD [150]. Tryptophan metabolism has also been linked with severity of IBD [151]. The enzyme indoleamine 2,3-dioxygenase-1 (IDO-1) is involved in the KYN pathway. It is upregulated in inflammatory environments leading to increased metabolism of Tryptophan to KYN, thereby reducing reserves needed for serotonin production. Research in mice models has shown increased KYN levels initiated by the increased corticosterone level in mice exposed to immobilisation stress. KYN has been identified as a risk factor for IBD in a recent two-sample Mendelian randomization study [152]. There is an implication that this is a potential relationship linking between anxiety/depression and IBD severity [153].

### 4.9. Roseburia

*Roseburia* is a genus of anaerobic Gram-positive, rod-shaped bacterium. It is a producer of SCFAs and its prevalence is inversely associated with IBD [154]. Luo et al. found treatment with *Roseburia intestinalis* (*R. intestinalis*) reduced levels of IL-17 and increased secretion of anti-inflammatory mediators such as IL-10 [154,155]. *R. intestinalis* has also been shown to reduce expression of gut serotonin levels and to alleviate depression-like symptoms in rodents [154,156]. There is a documented association of *Roseburia* depletion and multiple mental health disorders including depression [157]. However, Jiang et al. found conflicting outcomes where patients with MDD showed a higher abundance of *Roseburia* [76]. Further research is needed to draw conclusions on the role of *Roseburia* in this relationship.

### 4.10. Fusobacterium nucleatum (F. nucleatum)

*F*. *nucleatum* has been shown to be increased in the gut of patients diagnosed with IBD. This bacterium increases release of IL-6, IL-17 and induces intestinal inflammation [158]. *F. nucleatum* as well as other microbiota such as haemorrhagic *E. coli* possess receptors called Quorum sensing sensor kinase C (QseC) and Quorum sensing sensor kinase E (QseE). Both QseC and QseE have been shown to regulate virulence in haemorrhagic *E. coli* infection through their involvement in epithelial cell invasion and intramacrophage survival. They also demonstrated involvement in gene expression and colonisation in other bacterial species [159,160]. These receptors recognise host Noradrenaline (NA). NA is increased in chronic stress and its dysregulation has been reported in anxiety and depression [161].

One study in mice models found NA could assist *F. nucleatum* in invading the intestine and worsening colitis via noradrenaline’s upregulation of the Quorum sensing pathway [162]. Inhibition of QseC by using LED 209 (a small molecule) inhibited the effect of NA on QseC in *F. nucleatum* as well as reduced inflammation in IBD mice models.

*F. nucleatum* can therefore link stress with an effect on intestinal inflammation via NA’s activation of the quorum sensing pathway.

Other bacterial species such as *Klebsiella*, *Akkermansia* and *Prevotella* have been associated with anxiety/depressed mood and IBD, although this relationship is still not fully understood [163,164,165].

### 4.11. Diversity

A recent systematic review considered studies looking at microbiome diversity in patients with anxiety and depression. They used tools such as alpha (α-diversity) and beta diversity (β-diversity) to assess variations in the gut microbiome [148].

Alpha-diversity refers to the species diversity within a single community/location. Out of the 21 included studies, 16 showed no significant difference between alpha diversity in patients with depression and those deemed to be healthy. However, four of these studies showed a reported significant reduction in diversity in patients with depression when compared to healthy volunteers. Three also showed lower α-diversity in those with GAD vs. control groups. The review also examined β-diversity-a measure of inter-individual diversity in patients with a diagnosis of depression. Thirteen of the studies found a significant difference in gut microbiota β-diversity between patients with depression and healthy individuals.

Another study including 198 individuals found that patients diagnosed with anxiety also had a lower α-diversity [166]. This is further supported by the recent 2025 study that observed a significantly reduced α-diversity in patients with CD and a coexisting diagnosis of depression compared to those with just CD [150].

It has been demonstrated through varying levels of research that microbiome diversity is negatively correlated with IBD severity and that this improves in disease remission [167]. Thus, not only are the individual components of the microbiome targets for understanding this relationship, but the overall diversity could be the association underpinning this occurrence.

## 5. Discussion

This review highlights the complex bidirectional relationship between mental health conditions such as anxiety, depression and IBD. Anxiety and depression are becoming increasingly recognised as co-morbidities that not only influence outcomes in IBD but may even predispose to the new onset of IBD. However, this multi-dimensional neuro-endocrine-immune-microbial circuit is not yet fully understood.

The presence of anxiety and depression approximately double the odds of poor outcomes in IBD patients. These include increased risk of flare ups, steroid resistance, emergency department attendance, hospital admission, therapy escalation and surgical intervention [14,15,16]. Patients with concurrent IBD and mental health conditions who were offered mental health support not only report improved mental health outcomes but also higher total quality of life scores [19].

Despite cortisol’s role in immunosuppression, a sustained HPA axis activation such as in anxiety and depression appears to sustain gut inflammation, possibly via ‘glucocorticoid resistance’ or ‘pro-inflammatory cortisol’ models [25,26,27,28,29,30,31,32,33]. Another mechanism promoting inflammation is through the degranulation of mast cells following stimulation by CRH peripherally as a response to stressful stimuli [36,37,38,39,40]. They are commonly seen in both CD and UC and provide duality in their role; being both pro and anti-inflammatory. They can produce cytokines upon stimulation but also store anti-inflammatory components such as heparin [46,47,48]. Their deficiency seen in IL-10 deficient mice predisposes to IBD [50]. Their complex role in the pathogenesis of IBD requires further research, especially as their duality in function may be context dependent.

The GBA, when activated as a result of extended stress, also stimulates **e**nteric **g**lia **a**ssociated with **p**sychological **s**tress (eGAPS) in the ENS; promoting inflammation through the production of CSF1 and subsequently TNF release from mononuclear phagocytes [34]. This autonomic dysregulation as a result of stress also affects gut motility and intestinal permeability and can induce colitis [34,54,55,56,57,58,59].

The inconsistency of cytokine alterations between anxiety and depression suggests that deficits of anti-inflammatory cytokines like IL4/IL-10 may be disease specific [62,63,64,65]. Therefore, it is felt that management of IBD with co-morbid psychiatric conditions may benefit from tailored management according to the type of associated mental health disorder.

Antidepressants may be protective against the onset of IBD in patients with depression. SSRIs and TCAs were significantly protective against new onset CD more than UC. On the other hand, mirtazapine and SNRIs protect against UC more than CD [20]. Thereby implying that there may be distinct pathways linking psychiatric conditions and different IBD subtypes. Given the higher risk of poor outcomes in those suffering from anxiety and depression, psychological interventions and antidepressant use may mitigate this by reducing the burden of these mental health conditions. This would be through not only managing these mental health conditions but also improving disease course and prognosis [19,20]. For instance, when prescribing anti-depressants to CD patients, SSRIs or TCAs may become the preferred treatment over SNRIs or mirtazapine if no other contraindications exist. This, however, would require further validation studies.

Alterations in the gut microbiome are now known to be associated with mental health disorders and the inflammatory pathways seen in IBD [67,68,69,70,79,106]. They are also associated with disruptions to the BBB and susceptibility to neuroinflammation in the context of chronic colitis [91,95]. Both the GVB and the BBB share a similar architecture and thus it is important to note that systemic processes that affect one may also affect the other [92]. The interaction of alterations in gut microbiome appears to be bidirectional and influenced by a reduction in protective species (e.g., *Faecalibacterium*, *Bifidobacterium*) and increased prevalence of those that are pathogenic (e.g., *E. coli*) [103,104,105,109,110,111,112,113,114,115,116,117,118]. These changes coincide with reduced microbial diversity [79,106]. The microbiome influences serotonin metabolism, autophagy and production of metabolites such as SCFAs [81,86,87,88,89,98,99,100,101,102]. These link intestinal inflammation and mental health disorders. Certain species such as *B. vulgatus* or *C. butyricum* show strain-specific protective roles [139].

Thus far, the literature in this area is faced with several limitations, including the predominance of small cross-sectional studies, studies conducted in single populations and those with a strong reliance on animal models. Human trials remain limited, especially given the ethical considerations of chronic stress exposure.

There are several research gaps identified that merit further investigation:

1. High quality longitudinal cohort studies to explore the temporal association of anxiety and depression, and incidence and progression of IBD. Studies that can categorise severity of anxiety and depression would help to identify a dose effect.
2. Further data describing different antidepressant classes used in UC and CD with co-morbid anxiety and depression, and their effects on IBD outcomes.
3. The role of serotonergic microbiota in the development and progression of IBD and whether this represents a potential therapeutic opportunity [90].
4. Further research verifying whether a causal relationship exists between gut microbiome alterations, at a genus or species’ level, and new onset of IBD.
5. Exploring gut microbiome altering strategies including dietary modifications, probiotic use, faecal microbiota transplantation as potential bridges between gut and mental health [120,121,122,123,124,125,127].
6. There is a need for external validation of single-population study findings.

In conclusion, despite progress in understanding the pathogenesis, management, and prevention of IBD in relation to mental illness, further research is required. Given the rising prevalence of anxiety, depression, and IBD this represents an important opportunity to improve outcomes of both mental and physical health in this patient group.

## Data Availability

No new data were created or analysed in this study. Data sharing is not applicable to this article.

## References

[1] Kirsner J.B. (2001). Historical Origins of Current IBD Concepts. World J. Gastroenterol..

[2] Gracie D.J., Guthrie E.A., Hamlin P.J., Ford A.C. (2018). Bi-Directionality of Brain-Gut Interactions in Patients with Inflammatory Bowel Disease. Gastroenterology.

[3] Luo J., Xu Z., Noordam R., van Heemst D., Li-Gao R. (2022). Depression and Inflammatory Bowel Disease: A Bidirectional Two-Sample Mendelian Randomization Study. J. Crohns Colitis.

[4] Singh N., Bernstein C.N. (2022). Environmental Risk Factors for Inflammatory Bowel Disease. UEG J..

[5] Meuret A.E., Tunnell N., Roque A., Kim Y.-K. (2020). Anxiety Disorders and Medical Comorbidity: Treatment Implications. Anxiety Disorders.

[6] Țenea-Cojan Ș.-T., Dinescu V.-C., Gheorman V., Dragne I.-G., Gheorman V., Forțofoiu M.-C., Fortofoiu M., Dobrinescu A.G. (2025). Exploring Multidisciplinary Approaches to Comorbid Psychiatric and Medical Disorders: A Scoping Review. Life.

[7] American Psychiatric Association (2022). Diagnostic and Statistical Manual of Mental Disorders: DSM-5-TR.

[8] Otte C., Gold S.M., Penninx B.W., Pariante C.M., Etkin A., Fava M., Mohr D.C., Schatzberg A.F. (2016). Major Depressive Disorder. Nat. Rev. Dis. Primers.

[9] Bisgaard T.H., Allin K.H., Elmahdi R., Jess T. (2023). The Bidirectional Risk of Inflammatory Bowel Disease and Anxiety or Depression: A Systematic Review and Meta-Analysis. Gen. Hosp. Psychiatry.

[10] Piovani D., Armuzzi A., Bonovas S. (2024). Association of Depression with Incident Inflammatory Bowel Diseases: A Systematic Review and Meta-Analysis. Inflamm. Bowel Dis..

[11] Simon G.E., Moise N., Mohr D.C. (2024). Management of Depression in Adults: A Review. JAMA.

[12] Remes O., Brayne C., van der Linde R., Lafortune L. (2016). A Systematic Review of Reviews on the Prevalence of Anxiety Disorders in Adult Populations. Brain Behav..

[13] Walker J.R., Ediger J.P., Graff L.A., Greenfeld J.M., Clara I., Lix L., Rawsthorne P., Miller N., Rogala L., McPhail C.M. (2008). The Manitoba IBD Cohort Study: A Population-Based Study of the Prevalence of Lifetime and 12-Month Anxiety and Mood Disorders. Am. J. Gastroenterol..

[14] Ji Y., Li H., Dai G., Zhang X., Ju W. (2024). Systematic Review and Meta-Analysis: Impact of Depression on Prognosis in Inflammatory Bowel Disease. J. Gastroenterol. Hepatol..

[15] Duan S., Yang Y., Cao Y., Chen P., Liang C., Zhang Y. (2023). Symptoms of Anxiety and Depression Associated with Steroid Efficacy and Clinical Outcomes in Patients with Inflammatory Bowel Disease. Front. Psychiatry.

[16] Fairbrass K.M., Lovatt J., Barberio B., Yuan Y., Gracie D.J., Ford A.C. (2022). Bidirectional Brain-Gut Axis Effects Influence Mood and Prognosis in IBD: A Systematic Review and Meta-Analysis. Gut.

[17] Gao X., Tang Y., Lei N., Luo Y., Chen P., Liang C., Duan S., Zhang Y. (2021). Symptoms of Anxiety/Depression Is Associated with More Aggressive Inflammatory Bowel Disease. Sci. Rep..

[18] Moran G.W., Gordon M., Sinopolou V., Radford S.J., Darie A.-M., Vuyyuru S.K., Alrubaiy L., Arebi N., Blackwell J., Butler T.D. (2025). British Society of Gastroenterology Guidelines on Inflammatory Bowel Disease in Adults: 2025. Gut.

[19] Lores T., Goess C., Mikocka-Walus A., Collins K.L., Burke A.L.J., Chur-Hansen A., Delfabbro P., Andrews J.M. (2019). Integrated Psychological Care Is Needed, Welcomed and Effective in Ambulatory Inflammatory Bowel Disease Management: Evaluation of a New Initiative. J. Crohn’s Colitis.

[20] Frolkis A.D., Vallerand I.A., Shaheen A.-A., Lowerison M.W., Swain M.G., Barnabe C., Patten S.B., Kaplan G.G. (2019). Depression Increases the Risk of Inflammatory Bowel Disease, Which May Be Mitigated by the Use of Antidepressants in the Treatment of Depression. Gut.

[21] Ashton J.J., Beattie R.M. (2024). Inflammatory Bowel Disease: Recent Developments. Arch. Dis. Child..

[22] Peppas S., Pansieri C., Piovani D., Danese S., Peyrin-Biroulet L., Tsantes A.G., Brunetta E., Tsantes A.E., Bonovas S. (2021). The Brain-Gut Axis: Psychological Functioning and Inflammatory Bowel Diseases. J. Clin. Med..

[23] Abautret-Daly Á., Dempsey E., Parra-Blanco A., Medina C., Harkin A. (2018). Gut–Brain Actions Underlying Comorbid Anxiety and Depression Associated with Inflammatory Bowel Disease. Acta Neuropsychiatr..

[24] Collins S.M. (2020). Interrogating the Gut-Brain Axis in the Context of Inflammatory Bowel Disease: A Translational Approach. Inflamm. Bowel Dis..

[25] Sun Y., Li L., Xie R., Wang B., Jiang K., Cao H. (2019). Stress Triggers Flare of Inflammatory Bowel Disease in Children and Adults. Front. Pediatr..

[26] Pariante C.M., Miller A.H. (2001). Glucocorticoid Receptors in Major Depression: Relevance to Pathophysiology and Treatment. Biol. Psychiatry.

[27] Faravelli C., Sauro C.L., Godini L., Lelli L., Benni L., Pietrini F., Lazzeretti L., Talamba G.A., Fioravanti G., Ricca V. (2012). Childhood Stressful Events, HPA Axis and Anxiety Disorders. World J. Psychiatry.

[28] Gądek-Michalska A., Tadeusz J., Rachwalska P., Bugajski J. (2013). Cytokines, Prostaglandins and Nitric Oxide in the Regulation of Stress-Response Systems. Pharmacol. Rep..

[29] Gold P.W., Machado-Vieira R., Pavlatou M.G. (2015). Clinical and Biochemical Manifestations of Depression: Relation to the Neurobiology of Stress. Neural Plast..

[30] Amasi-Hartoonian N., Sforzini L., Cattaneo A., Pariante C.M. (2022). Cause or Consequence? Understanding the Role of Cortisol in the Increased Inflammation Observed in Depression. Curr. Opin. Endocr. Metab. Res..

[31] Brattsand R., Linden M. (1996). Cytokine Modulation by Glucocorticoids: Mechanisms and Actions in Cellular Studies. Aliment. Pharmacol. Ther..

[32] Lamberts S. (1996). The Glucocorticoid Insensitivity Syndrome. Horm. Res..

[33] Pariante C.M., Lightman S.L. (2008). The HPA Axis in Major Depression: Classical Theories and New Developments. Trends Neurosci..

[34] Schneider K.M., Blank N., Alvarez Y., Thum K., Lundgren P., Litichevskiy L., Sleeman M., Bahnsen K., Kim J., Kardo S. (2023). The Enteric Nervous System Relays Psychological Stress to Intestinal Inflammation. Cell.

[35] Stengel A., Taché Y. (2010). Corticotropin-Releasing Factor Signaling and Visceral Response to Stress. Exp. Biol. Med..

[36] La Fleur S.E., Wick E.C., Idumalla P.S., Grady E.F., Bhargava A. (2005). Role of Peripheral Corticotropin-Releasing Factor and Urocortin II in Intestinal Inflammation and Motility in Terminal Ileum. Proc. Natl. Acad. Sci. USA.

[37] Kawahito Y., Sano H., Kawata M., Yuri K., Mukai S., Yamamura Y., Kato H., Chrousos G.P., Wilder R.L., Kondo M. (1994). Local Secretion of Corticotropin-Releasing Hormone by Enterochromaffin Cells in Human Colon. Gastroenterology.

[38] Tache Y., Larauche M., Yuan P.-Q., Million M. (2018). Brain and Gut CRF Signaling: Biological Actions and Role in the Gastrointestinal Tract. Curr. Mol. Pharmacol..

[39] Vanuytsel T., van Wanrooy S., Vanheel H., Vanormelingen C., Verschueren S., Houben E., Rasoel S.S., Tόth J., Holvoet L., Farré R. (2014). Psychological Stress and Corticotropin-Releasing Hormone Increase Intestinal Permeability in Humans by a Mast Cell-Dependent Mechanism. Gut.

[40] Tache Y., Perdue M. (2004). Role of Peripheral CRF Signalling Pathways in Stress-related Alterations of Gut Motility and Mucosal Function. Neurogastroenterol. Motil..

[41] Bear T., Dalziel J., Coad J., Roy N., Butts C., Gopal P. (2021). The Microbiome-Gut-Brain Axis and Resilience to Developing Anxiety or Depression under Stress. Microorganisms.

[42] La Torre D., Van Oudenhove L., Vanuytsel T., Verbeke K. (2023). Psychosocial Stress-Induced Intestinal Permeability in Healthy Humans: What Is the Evidence?. Neurobiol. Stress.

[43] Karban A., Eliakim R. (2007). Effect of Smoking on Inflammatory Bowel Disease: Is It Disease or Organ Specific?. World J. Gastroenterol..

[44] Overman E.L., Rivier J.E., Moeser A.J. (2012). CRF Induces Intestinal Epithelial Barrier Injury via the Release of Mast Cell Proteases and TNF-α. PLoS ONE.

[45] Wallon C., Persborn M., Jönsson M., Wang A., Phan V., Lampinen M., Vicario M., Santos J., Sherman P.M., Carlson M. (2011). Eosinophils Express Muscarinic Receptors and Corticotropin-Releasing Factor to Disrupt the Mucosal Barrier in Ulcerative Colitis. Gastroenterology.

[46] He S.-H. (2004). Key Role of Mast Cells and Their Major Secretory Products in Inflammatory Bowel Disease. World J. Gastroenterol..

[47] Dvorak A.M., Monahan R.A., Osage J.E., Dickersin G.R. (1980). Crohn’s Disease: Transmission Electron Microscopic Studies: II. Immunologic Inflammatory Response. Alterations of Mast Cells, Basophils, Eosinophils, and the Microvasculature. Hum. Pathol..

[48] Lloyd G., Green F., Fox H., Mani V., Turnberg L. (1975). Mast Cells and Immunoglobulin E in Inflammatory Bowel Disease. Gut.

[49] Aljameeli A.M., Alsuwayt B., Bharati D., Gohri V., Mohite P., Singh S., Chidrawar V. (2025). Chloride Channels and Mast Cell Function: Pioneering New Frontiers in IBD Therapy. Mol. Cell Biochem..

[50] Chichlowski M., Westwood G.S., Abraham S.N., Hale L.P. (2010). Role of Mast Cells in Inflammatory Bowel Disease and Inflammation-Associated Colorectal Neoplasia in IL-10-Deficient Mice. PLoS ONE.

[51] He Y., Chen C.-L., He J., Liu S.-D. (2023). Causal Associations Between Inflammatory Bowel Disease and Anxiety: A Bidirectional Mendelian Randomization Study. World J. Gastroenterol..

[52] Carabotti M., Scirocco A., Maselli M.A., Severi C. (2015). The Gut-Brain Axis: Interactions Between Enteric Microbiota, Central and Enteric Nervous Systems. Ann. Gastroenterol..

[53] Rao M., Gershon M.D. (2016). The Bowel and beyond: The Enteric Nervous System in Neurological Disorders. Nat. Rev. Gastroenterol. Hepatol..

[54] Johnson J., Campisi J., Sharkey C., Kennedy S., Nickerson M., Greenwood B., Fleshner M. (2005). Catecholamines Mediate Stress-Induced Increases in Peripheral and Central Inflammatory Cytokines. Neuroscience.

[55] Furness J.B. (2009). Enteric Nervous System. Encyclopedia of Neuroscience.

[56] Sharkey K.A., Mawe G.M. (2023). The Enteric Nervous System. Physiol. Rev..

[57] Goyal R.K., Hirano I. (1996). The Enteric Nervous System. N. Engl. J. Med..

[58] Gershon M.D. (1981). The Enteric Nervous System. Annu. Rev. Neurosci..

[59] Won E., Kim Y.-K. (2016). Stress, the Autonomic Nervous System, and the Immune-Kynurenine Pathway in the Etiology of Depression. Curr. Neuropharmacol..

[60] Billiet T., Rutgeerts P., Ferrante M., Van Assche G., Vermeire S. (2014). Targeting TNF-α for the Treatment of Inflammatory Bowel Disease. Expert Opin. Biol. Ther..

[61] Targan S.R., Hanauer S.B., Van Deventer S.J.H., Mayer L., Present D.H., Braakman T., DeWoody K.L., Schaible T.F., Rutgeerts P.J. (1997). A Short-Term Study of Chimeric Monoclonal Antibody cA2 to Tumor Necrosis Factor α for Crohn’s Disease. N. Engl. J. Med..

[62] Neurath M.F. (2014). Cytokines in Inflammatory Bowel Disease. Nat. Rev. Immunol..

[63] Costello H., Gould R.L., Abrol E., Howard R. (2019). Systematic Review and Meta-Analysis of the Association Between Peripheral Inflammatory Cytokines and Generalised Anxiety Disorder. BMJ Open.

[64] Dowlati Y., Herrmann N., Swardfager W., Liu H., Sham L., Reim E.K., Lanctôt K.L. (2010). A Meta-Analysis of Cytokines in Major Depression. Biol. Psychiatry.

[65] Vieira M.M., Ferreira T.B., Pacheco P.A., Barros P.O., Almeida C.R., Araújo-Lima C.F., Silva-Filho R.G., Hygino J., Andrade R.M., Linhares U.C. (2010). Enhanced Th17 Phenotype in Individuals with Generalized Anxiety Disorder. J. Neuroimmunol..

[66] Hou R., Garner M., Holmes C., Osmond C., Teeling J., Lau L., Baldwin D.S. (2017). Peripheral Inflammatory Cytokines and Immune Balance in Generalised Anxiety Disorder: Case-Controlled Study. Brain Behav. Immun..

[67] Halfvarson J., Brislawn C.J., Lamendella R., Vázquez-Baeza Y., Walters W.A., Bramer L.M., D’Amato M., Bonfiglio F., McDonald D., Gonzalez A. (2017). Dynamics of the Human Gut Microbiome in Inflammatory Bowel Disease. Nat. Microbiol..

[68] Sokol H., Pigneur B., Watterlot L., Lakhdari O., Bermúdez-Humarán L.G., Gratadoux J.-J., Blugeon S., Bridonneau C., Furet J.-P., Corthier G. (2008). *Faecalibacterium prausnitzii* Is an Anti-Inflammatory Commensal Bacterium Identified by Gut Microbiota Analysis of Crohn Disease Patients. Proc. Natl. Acad. Sci. USA.

[69] Willing B., Halfvarson J., Dicksved J., Rosenquist M., Järnerot G., Engstrand L., Tysk C., Jansson J.K. (2009). Twin Studies Reveal Specific Imbalances in the Mucosa-Associated Microbiota of Patients with Ileal Crohn’s Disease. Inflamm. Bowel Dis..

[70] Willing B.P., Dicksved J., Halfvarson J., Andersson A.F., Lucio M., Zheng Z., Järnerot G., Tysk C., Jansson J.K., Engstrand L. (2010). A Pyrosequencing Study in Twins Shows That Gastrointestinal Microbial Profiles Vary with Inflammatory Bowel Disease Phenotypes. Gastroenterology.

[71] Karen C., Shyu D.J.H., Rajan K.E. (2021). *Lactobacillus paracasei* Supplementation Prevents Early Life Stress-Induced Anxiety and Depressive-Like Behavior in Maternal Separation Model-Possible Involvement of Microbiota-Gut-Brain Axis in Differential Regulation of MicroRNA124a/132 and Glutamate Receptors. Front. Neurosci..

[72] Steenbergen L., Sellaro R., van Hemert S., Bosch J.A., Colzato L.S. (2015). A Randomized Controlled Trial to Test the Effect of Multispecies Probiotics on Cognitive Reactivity to Sad Mood. Brain Behav. Immun..

[73] Zhang F., Qi N., Zeng Y., Bao M., Chen Y., Liao J., Wei L., Cao D., Huang S., Luo Q. (2020). The Endogenous Alterations of the Gut Microbiota and Feces Metabolites Alleviate Oxidative Damage in the Brain of LanCL1 Knockout Mice. Front. Microbiol..

[74] Lin S., Li Q., Jiang S., Xu Z., Jiang Y., Liu L., Jiang J., Tong Y., Wang P. (2021). Crocetin Ameliorates Chronic Restraint Stress-Induced Depression-like Behaviors in Mice by Regulating MEK/ERK Pathways and Gut Microbiota. J. Ethnopharmacol..

[75] Walker W.A. (2017). Dysbiosis. The Microbiota in Gastrointestinal Pathophysiology.

[76] Jiang H., Ling Z., Zhang Y., Mao H., Ma Z., Yin Y., Wang W., Tang W., Tan Z., Shi J. (2015). Altered Fecal Microbiota Composition in Patients with Major Depressive Disorder. Brain Behav. Immun..

[77] Honda K., Littman D.R. (2016). The Microbiota in Adaptive Immune Homeostasis and Disease. Nature.

[78] Palmela C., Chevarin C., Xu Z., Torres J., Sevrin G., Hirten R., Barnich N., Ng S.C., Colombel J.-F. (2018). Adherent-Invasive *Escherichia coli* in Inflammatory Bowel Disease. Gut.

[79] Carvalho F.A., Barnich N., Sauvanet P., Darcha C., Gelot A., Darfeuille-Michaud A. (2008). Crohn’s Disease-Associated *Escherichia coli* LF82 Aggravates Colitis in Injured Mouse Colon via Signaling by Flagellin. Inflamm. Bowel Dis..

[80] Haq S., Wang H., Grondin J., Banskota S., Marshall J.K., Khan I.I., Chauhan U., Cote F., Kwon Y.H., Philpott D. (2021). Disruption of Autophagy by Increased 5-HT Alters Gut Microbiota and Enhances Susceptibility to Experimental Colitis and Crohn’s Disease. Sci. Adv..

[81] Lin J., Liu W., Guan J., Cui J., Shi R., Wang L., Chen D., Liu Y. (2023). Latest Updates on the Serotonergic System in Depression and Anxiety. Front. Synaptic Neurosci..

[82] Berger M., Gray J.A., Roth B.L. (2009). The Expanded Biology of Serotonin. Annu. Rev. Med..

[83] Chen M., Gao L., Chen P., Feng D., Jiang Y., Chang Y., Jin J., Chu F.-F., Gao Q. (2016). Serotonin-Exacerbated DSS-Induced Colitis Is Associated with Increase in MMP-3 and MMP-9 Expression in the Mouse Colon. Mediat. Inflamm..

[84] Kwon Y.H., Wang H., Denou E., Ghia J.-E., Rossi L., Fontes M.E., Bernier S.P., Shajib M.S., Banskota S., Collins S.M. (2019). Modulation of Gut Microbiota Composition by Serotonin Signaling Influences Intestinal Immune Response and Susceptibility to Colitis. Cell. Mol. Gastroenterol. Hepatol..

[85] Ghia J., Li N., Wang H., Collins M., Deng Y., El–Sharkawy R.T., Côté F., Mallet J., Khan W.I. (2009). Serotonin Has a Key Role in Pathogenesis of Experimental Colitis. Gastroenterology.

[86] Grondin J.A., Khan W.I. (2024). Emerging Roles of Gut Serotonin in Regulation of Immune Response, Microbiota Composition and Intestinal Inflammation. J. Can. Assoc. Gastroenterol..

[87] Tsuboi K., Nishitani M., Takakura A., Imai Y., Komatsu M., Kawashima H. (2015). Autophagy Protects against Colitis by the Maintenance of Normal Gut Microflora and Secretion of Mucus*. J. Biol. Chem..

[88] Saitoh T., Fujita N., Jang M.H., Uematsu S., Yang B.-G., Satoh T., Omori H., Noda T., Yamamoto N., Komatsu M. (2008). Loss of the Autophagy Protein Atg16L1 Enhances Endotoxin-Induced IL-1β Production. Nature.

[89] Haq S., Grondin J., Banskota S., Khan W.I. (2019). Autophagy: Roles in Intestinal Mucosal Homeostasis and Inflammation. J. Biomed. Sci..

[90] Sanidad K.Z., Rager S.L., Carrow H.C., Ananthanarayanan A., Callaghan R., Hart L.R., Li T., Ravisankar P., Brown J.A., Amir M. (2024). Gut Bacteria–Derived Serotonin Promotes Immune Tolerance in Early Life. Sci. Immunol..

[91] Braniste V., Al-Asmakh M., Kowal C., Anuar F., Abbaspour A., Tóth M., Korecka A., Bakocevic N., Ng L.G., Kundu P. (2014). The gut microbiota influences blood-brain barrier permeability in mice. Sci. Transl. Med..

[92] Scalise A.A., Kakogiannos N., Zanardi F., Iannelli F., Giannotta M. (2021). The Blood–Brain and Gut–Vascular Barriers: From the Perspective of Claudins. Tissue Barriers.

[93] Tommaso N.D., Santopaolo F., Gasbarrini A., Ponziani F.R. (2023). The Gut–Vascular Barrier as a New Protagonist in Intestinal and Extraintestinal Diseases. Int. J. Mol. Sci..

[94] Vitali R., Stronati L., Negroni A., Di Nardo G., Pierdomenico M., Del Giudice E., Rossi P., Cucchiara S. (2011). Fecal HMGB1 Is a Novel Marker of Intestinal Mucosal Inflammation in Pediatric Inflammatory Bowel Disease. Am. J. Gastroenterol..

[95] Mitchell J., Kim S.J., Howe C., Lee S., Her J.Y., Patel M., Kim G., Lee J., Im E., Rhee S.H. (2022). Chronic Intestinal Inflammation Suppresses Brain Activity by Inducing Neuroinflammation in Mice. Am. J. Pathol..

[96] Sheline Y.I., Sanghavi M., Mintun M.A., Gado M.H. (1999). Depression duration but not age predicts hippocampal volume loss in medically healthy women with recurrent major depression. J. Neurosci..

[97] Videbech P., Ravnkilde B. (2004). Hippocampal Volume and Depression: A Meta-Analysis of MRI Studies. Am. J. Psychiatry.

[98] Strandwitz P. (2018). Neurotransmitter Modulation by the Gut Microbiota. Brain Res..

[99] Koopman N., Katsavelis D., Hove A.S.T., Brul S., de Jonge W.J., Seppen J. (2021). The Multifaceted Role of Serotonin in Intestinal Homeostasis. Int. J. Mol. Sci..

[100] González Delgado S., Garza-Veloz I., Trejo-Vazquez F., Martinez-Fierro M.L. (2022). Interplay Between Serotonin, Immune Response, and Intestinal Dysbiosis in Inflammatory Bowel Disease. Int. J. Mol. Sci..

[101] Reigstad C.S., Salmonson C.E., Rainey J.F., Szurszewski J.H., Linden D.R., Sonnenburg J.L., Farrugia G., Kashyap P.C. (2015). Gut Microbes Promote Colonic Serotonin Production through an Effect of Short-Chain Fatty Acids on Enterochromaffin Cells. FASEB J..

[102] Han A., Sung Y.-B., Chung S.-Y., Kwon M.-S. (2014). Possible Additional Antidepressant-like Mechanism of Sodium Butyrate: Targeting the Hippocampus. Neuropharmacology.

[103] Mimouna S., Gonçalvès D., Barnich N., Darfeuille-Michaud A., Hofman P., Vouret-Craviari V. (2011). Crohn Disease-Associated *Escherichia coli* Promote Gastrointestinal Inflammatory Disorders by Activation of HIF-Dependent Responses. Gut Microbes.

[104] Jang H.-M., Lee K.-E., Lee H.-J., Kim D.-H. (2018). Immobilization Stress-Induced *Escherichia coli* Causes Anxiety by Inducing NF-κB Activation through Gut Microbiota Disturbance. Sci. Rep..

[105] Lee M., Chang E.B. (2021). Inflammatory Bowel Diseases and the Microbiome: Searching the Crime Scene for Clues. Gastroenterology.

[106] Leylabadlo H.E., Ghotaslou R., Feizabadi M.M., Farajnia S., Moaddab S.Y., Ganbarov K., Khodadadi E., Tanomand A., Sheykhsaran E., Yousefi B. (2020). The Critical Role of *Faecalibacterium prausnitzii* in Human Health: An Overview. Microb. Pathog..

[107] Chen Y., Bai J., Wu D., Yu S., Qiang X., Bai H., Wang H., Peng Z. (2019). Association Between Fecal Microbiota and Generalized Anxiety Disorder: Severity and Early Treatment Response. J. Affect. Disord..

[108] Rondepierre F., Meynier M., Gagniere J., Deneuvy V., Deneuvy A., Roche G., Baudu E., Pereira B., Bonnet R., Barnich N. (2024). Preclinical and Clinical Evidence of the Association of Colibactin-Producing *Escherichia coli* with Anxiety and Depression in Colon Cancer. World J. Gastroenterol..

[109] Lopez-Siles M., Duncan S.H., Garcia-Gil L.J., Martinez-Medina M. (2017). *Faecalibacterium prausnitzii*: From Microbiology to Diagnostics and Prognostics. ISME J..

[110] Miquel S., Martín R., Rossi O., Bermúdez-Humarán L., Chatel J., Sokol H., Thomas M., Wells J., Langella P. (2013). *Faecalibacterium prausnitzii* and Human Intestinal Health. Curr. Opin. Microbiol..

[111] Inan M.S., Rasoulpour R.J., Yin L., Hubbard A.K., Rosenberg D.W., Giardina C. (2000). The Luminal Short-Chain Fatty Acid Butyrate Modulates NF-κB Activity in a Human Colonic Epithelial Cell Line. Gastroenterology.

[112] Jansson J., Willing B., Lucio M., Fekete A., Dicksved J., Halfvarson J., Tysk C., Schmitt-Kopplin P. (2009). Metabolomics Reveals Metabolic Biomarkers of Crohn’s Disease. PLoS ONE.

[113] Sokol H., Seksik P., Furet J.P., Firmesse O., Nion-Larmurier I., Beaugerie L., Cosnes J., Corthier G., Marteau P., Doré J. (2009). Low Counts of *Faecalibacterium prausnitzii* in Colitis Microbiota. Inflamm. Bowel Dis..

[114] Klampfer L., Huang J., Sasazuki T., Shirasawa S., Augenlicht L. (2003). Inhibition of Interferon Gamma Signaling by the Short Chain Fatty Acid Butyrate. Mol. Cancer Res..

[115] Parada Venegas D., De la Fuente M.K., Landskron G., González M.J., Quera R., Dijkstra G., Harmsen H.J.M., Faber K.N., Hermoso M.A. (2019). Short Chain Fatty Acids (SCFAs)-Mediated Gut Epithelial and Immune Regulation and Its Relevance for Inflammatory Bowel Diseases. Front. Immunol..

[116] Samuthpongtorn C., Chan A.A., Ma W., Wang F., Nguyen L.H., Wang D.D., Okereke O.I., Huttenhower C., Chan A.T., Mehta R.S. (2024). *F. prausnitzii* Potentially Modulates the Association Between Citrus Intake and Depression. Microbiome.

[117] Martín R., Miquel S., Chain F., Natividad J.M., Jury J., Lu J., Sokol H., Theodorou V., Bercik P., Verdu E.F. (2015). *Faecalibacterium prausnitzii* Prevents Physiological Damages in a Chronic Low-Grade Inflammation Murine Model. BMC Microbiol..

[118] Hao Z., Wang W., Guo R., Liu H. (2019). *Faecalibacterium prausnitzii* (ATCC 27766) Has Preventive and Therapeutic Effects on Chronic Unpredictable Mild Stress-Induced Depression-like and Anxiety-like Behavior in Rats. Psychoneuroendocrinology.

[119] Kim C.-S., Shin G.-E., Cheong Y., Shin J.-H., Shin D.-M., Chun W.Y. (2022). Experiencing Social Exclusion Changes Gut Microbiota Composition. Transl. Psychiatry.

[120] Ganji-Arjenaki M., Rafieian-Kopaei M. (2018). Probiotics Are a Good Choice in Remission of Inflammatory Bowel Diseases: A Meta Analysis and Systematic Review. J. Cell Physiol..

[121] Heeney D.D., Gareau M.G., Marco M.L. (2018). Intestinal Lactobacillus in Health and Disease, a Driver or Just along for the Ride?. Curr. Opin. Biotechnol..

[122] Wang W., Chen L., Zhou R., Wang X., Song L., Huang S., Wang G., Xia B. (2014). Increased Proportions of Bifidobacterium and the Lactobacillus Group and Loss of Butyrate-Producing Bacteria in Inflammatory Bowel Disease. J. Clin. Microbiol..

[123] Lewis J.D., Chen E.Z., Baldassano R.N., Otley A.R., Griffiths A.M., Lee D., Bittinger K., Bailey A., Friedman E.S., Hoffmann C. (2015). Inflammation, Antibiotics, and Diet as Environmental Stressors of the Gut Microbiome in Pediatric Crohn’s Disease. Cell Host Microbe.

[124] Chen C.-M., Wu C.-C., Huang C.-L., Chang M.-Y., Cheng S.-H., Lin C.-T., Tsai Y.-C. (2022). *Lactobacillus plantarum* PS128 Promotes Intestinal Motility, Mucin Production, and Serotonin Signaling in Mice. Probiotics Antimicrob. Proteins.

[125] Yuan X., Chen B., Duan Z., Xia Z., Ding Y., Chen T., Liu H., Wang B., Yang B., Wang X. (2021). Depression and Anxiety in Patients with Active Ulcerative Colitis: Crosstalk of Gut Microbiota, Metabolomics and Proteomics. Gut Microbes.

[126] Barros-Santos T., Silva K.S.O., Libarino-Santos M., Cata-Preta E.G., Reis H.S., Tamura E.K., de Oliveira-Lima A.J., Berro L.F., Uetanabaro A.P.T., Marinho E.A.V. (2020). Effects of Chronic Treatment with New Strains of *Lactobacillus plantarum* on Cognitive, Anxiety- and Depressive-like Behaviors in Male Mice. PLoS ONE.

[127] Li C., Peng K., Xiao S., Long Y., Yu Q. (2023). The Role of Lactobacillus in Inflammatory Bowel Disease: From Actualities to Prospects. Cell Death Discov..

[128] Bien J., Palagani V., Bozko P. (2013). The Intestinal Microbiota Dysbiosis and Clostridium Difficile Infection: Is There a Relationship with Inflammatory Bowel Disease?. Ther. Adv. Gastroenterol..

[129] Nitzan O., Elias M., Chazan B., Raz R., Saliba W. (2013). Clostridium Difficile and Inflammatory Bowel Disease: Role in Pathogenesis and Implications in Treatment. World J. Gastroenterol..

[130] Joo M.-K., Ma X., Shin J.-W., Shin Y.-J., Kim D.-H. (2025). *Lactococcus Lactis* and *Bifidobacterium longum* Attenuate *Clostridioides difficile*- or *Clostridium symbiosum*-Induced Colitis and Depression/Anxiety-like Behavior in Male Mice. Microbes Infect..

[131] Usui N., Matsuzaki H., Shimada S. (2021). Characterization of Early Life Stress-Affected Gut Microbiota. Brain Sci..

[132] Bailey M.T., Dowd S.E., Galley J.D., Hufnagle A.R., Allen R.G., Lyte M. (2011). Exposure to a Social Stressor Alters the Structure of the Intestinal Microbiota: Implications for Stressor-Induced Immunomodulation. Brain Behav. Immun..

[133] Sun J., Wang F., Hu X., Yang C., Xu H., Yao Y., Liu J. (2018). *Clostridium butyricum* Attenuates Chronic Unpredictable Mild Stress-Induced Depressive-Like Behavior in Mice via the Gut-Brain Axis. J. Agric. Food Chem..

[134] Shah R., Cope J.L., Nagy-Szakal D., Dowd S., Versalovic J., Hollister E.B., Kellermayer R. (2016). Composition and Function of the Pediatric Colonic Mucosal Microbiome in Untreated Patients with Ulcerative Colitis. Gut Microbes.

[135] Mason B.L., Li Q., Minhajuddin A., Czysz A.H., Coughlin L.A., Hussain S.K., Koh A.Y., Trivedi M.H. (2020). Reduced Anti-Inflammatory Gut Microbiota Are Associated with Depression and Anhedonia. J. Affect. Disord..

[136] Zhou Y., Zhi F. (2016). Lower Level of Bacteroides in the Gut Microbiota Is Associated with Inflammatory Bowel Disease: A Meta-Analysis. BioMed Res. Int..

[137] Zhang Y., Fan Q., Hou Y., Zhang X., Yin Z., Cai X., Wei W., Wang J., He D., Wang G. (2022). Bacteroides Species Differentially Modulate Depression-like Behavior via Gut-Brain Metabolic Signaling. Brain Behav. Immun..

[138] Hu X., Li Y., Wu J., Zhang H., Huang Y., Tan X., Wen L., Zhou X., Xie P., Olasunkanmi O.I. (2023). Changes of Gut Microbiota Reflect the Severity of Major Depressive Disorder: A Cross Sectional Study. Transl. Psychiatry.

[139] Wu X., Xu J., Li J., Deng M., Shen Z., Nie K., Luo W., Zhang C., Ma K., Chen X. (2023). Bacteroides Vulgatus Alleviates Dextran Sodium Sulfate-Induced Colitis and Depression-like Behaviour by Facilitating Gut-Brain Axis Balance. Front. Microbiol..

[140] Liu Y., Hou Y., Wang G., Zheng X., Hao H. (2020). Gut Microbial Metabolites of Aromatic Amino Acids as Signals in Host–Microbe Interplay. Trends Endocrinol. Metab..

[141] Shao Y., Li Y., Xie S., Zhang B., Li J., Wang Y., Gao Y., Ma J., Kishawy A.T.Y., Gu L. (2025). Effects of 4-Hydroxyphenylacetic Acid, a Phenolic Acid Compound from *Yucca schidigera* Extract, on Immune Function and Intestinal Health in Meat Pigeons. Poult. Sci..

[142] Yao S., Zhao Z., Wang W., Liu X. (2021). *Bifidobacterium longum*: Protection against Inflammatory Bowel Disease. J. Immunol. Res..

[143] Jiang Y., Qu Y., Shi L., Ou M., Du Z., Zhou Z., Zhou H., Zhu H. (2024). The Role of Gut Microbiota and Metabolomic Pathways in Modulating the Efficacy of SSRIs for Major Depressive Disorder. Transl. Psychiatry.

[144] Zhang M., Zhou L., Zhang S., Yang Y., Xu L., Hua Z., Zou X. (2017). *Bifidobacterium longum* Affects the Methylation Level of Forkhead Box P3 Promoter in 2, 4, 6-Trinitrobenzenesulphonic Acid Induced Colitis in Rats. Microb. Pathog..

[145] Li Z., Peng C., Sun Y., Zhang T., Feng C., Zhang W., Huang T., Yao G., Zhang H., He Q. (2024). Both Viable *Bifidobacterium longum* Subsp. Infantis B8762 and Heat-Killed Cells Alleviate the Intestinal Inflammation of DSS-Induced IBD Rats. Microbiol. Spectr..

[146] Chen X., Fu Y., Wang L., Qian W., Zheng F., Hou X. (2019). *Bifidobacterium longum* and VSL#3^®^ Amelioration of TNBS-Induced Colitis Associated with Reduced HMGB1 and Epithelial Barrier Impairment. Dev. Comp. Immunol..

[147] Pinto-Sanchez M.I., Hall G.B., Ghajar K., Nardelli A., Bolino C., Lau J.T., Martin F.-P., Cominetti O., Welsh C., Rieder A. (2017). Probiotic *Bifidobacterium longum* NCC3001 Reduces Depression Scores and Alters Brain Activity: A Pilot Study in Patients with Irritable Bowel Syndrome. Gastroenterology.

[148] Cao Y., Cheng Y., Pan W., Diao J., Sun L., Meng M. (2025). Gut Microbiota Variations in Depression and Anxiety: A Systematic Review. BMC Psychiatry.

[149] Fang Z., Pan T., Li L., Wang H., Zhu J., Zhang H., Zhao J., Chen W., Lu W. (2022). *Bifidobacterium longum* Mediated Tryptophan Metabolism to Improve Atopic Dermatitis via the Gut-Skin Axis. Gut Microbes.

[150] Lin Y., Wang F.-T., Xia K., Gao R.-Y., Jiao Y.-R., Fang M., Chen C.-Q. (2025). Impact of Terminal Ileal Microbiota Dysbiosis and Tryptophan Metabolism Alterations on Mental Disorders in Patients with Crohn’s Disease. BMC Gastroenterol..

[151] Nikolaus S., Schulte B., Al-Massad N., Thieme F., Schulte D.M., Bethge J., Rehman A., Tran F., Aden K., Häsler R. (2017). Increased Tryptophan Metabolism Is Associated with Activity of Inflammatory Bowel Diseases. Gastroenterology.

[152] Yu F., Du Y., Li C., Zhang H., Lai W., Li S., Ye Z., Fu W., Li S., Li X.-G. (2024). Association Between Metabolites in Tryptophan-Kynurenine Pathway and Inflammatory Bowel Disease: A Two-Sample Mendelian Randomization. Sci. Rep..

[153] Notarangelo F.M., Schwarcz R. (2016). Restraint Stress During Pregnancy Rapidly Raises Kynurenic Acid Levels in Mouse Placenta and Fetal Brain. Dev. Neurosci..

[154] Nie K., Ma K., Luo W., Shen Z., Yang Z., Xiao M., Tong T., Yang Y., Wang X. (2021). Roseburia Intestinalis: A Beneficial Gut Organism from the Discoveries in Genus and Species. Front. Cell Infect. Microbiol..

[155] Zhu C., Song K., Shen Z., Quan Y., Tan B., Luo W., Wu S., Tang K., Yang Z., Wang X. (2018). Roseburia Intestinalis Inhibits Interleukin-17 Excretion and Promotes Regulatory T Cells Differentiation in Colitis. Mol. Med. Rep..

[156] Xu F., Cheng Y., Ruan G., Fan L., Tian Y., Xiao Z., Chen D., Wei Y. (2021). New Pathway Ameliorating Ulcerative Colitis: Focus on Roseburia Intestinalis and the Gut–Brain Axis. Ther. Adv. Gastroenterol..

[157] Li J., Ma Y., Bao Z., Gui X., Li A.N., Yang Z., Li M.D. (2020). Clostridiales Are Predominant Microbes That Mediate Psychiatric Disorders. J. Psychiatr. Res..

[158] Liu H., Hong X.L., Sun T.T., Huang X.W., Wang J.L., Xiong H. (2020). Fusobacterium Nucleatum Exacerbates Colitis by Damaging Epithelial Barriers and Inducing Aberrant Inflammation. J. Dig. Dis..

[159] Moreira C.G., Sperandio V. (2012). Interplay Between the QseC and QseE Bacterial Adrenergic Sensor Kinases in Salmonella Enterica Serovar Typhimurium Pathogenesis. Infect. Immun..

[160] Hughes D.T., Clarke M.B., Yamamoto K., Rasko D.A., Sperandio V. (2009). The QseC Adrenergic Signaling Cascade in Enterohemorrhagic *E. coli* (EHEC). PLOS Pathog..

[161] Ressler K.J., Nemeroff C.B. (2000). Role of Serotonergic and Noradrenergic Systems in the Pathophysiology of Depression and Anxiety Disorders. Depress. Anxiety.

[162] Zhang L., Chen G., Zeng X., Yue H., Zheng Q., Hu Q., Tian Q., Liang L., Zhao X., Yang Z. (2024). The Norepinephrine-QseC Axis Aggravates F. Nucleatum-Associated Colitis Through Interkingdom Signaling. Inflamm. Bowel Dis..

[163] Xiong R.-G., Li J., Cheng J., Zhou D.-D., Wu S.-X., Huang S.-Y., Saimaiti A., Yang Z.-J., Gan R.-Y., Li H.-B. (2023). The Role of Gut Microbiota in Anxiety, Depression, and Other Mental Disorders as Well as the Protective Effects of Dietary Components. Nutrients.

[164] Zhao X., Xu J., Wu D., Chen N., Liu Y. (2025). Gut Microbiota in Different Treatment Response Types of Crohn’s Disease Patients Treated with Biologics over a Long Disease Course. Biomedicines.

[165] Lin P., Ding B., Feng C., Yin S., Zhang T., Qi X., Lv H., Guo X., Dong K., Zhu Y. (2017). Prevotella and Klebsiella Proportions in Fecal Microbial Communities Are Potential Characteristic Parameters for Patients with Major Depressive Disorder. J. Affect. Disord..

[166] Malan-Müller S., Valles-Colomer M., Palomo T., Leza J.C. (2023). The Gut-Microbiota-Brain Axis in a Spanish Population in the Aftermath of the COVID-19 Pandemic: Microbiota Composition Linked to Anxiety, Trauma, and Depression Profiles. Gut Microbes.

[167] Gong D., Gong X., Wang L., Yu X., Dong Q. (2016). Involvement of Reduced Microbial Diversity in Inflammatory Bowel Disease. Gastroenterol. Res. Pract..

